# Cellulosic biofuel production using emulsified simultaneous saccharification and fermentation (eSSF) with conventional and thermotolerant yeasts

**DOI:** 10.1186/s13068-021-02008-7

**Published:** 2021-07-17

**Authors:** Shannon M. Hoffman, Maria Alvarez, Gilad Alfassi, Dmitry M. Rein, Sergio Garcia-Echauri, Yachin Cohen, José L. Avalos

**Affiliations:** 1 Department of Chemical and Biological Engineering, Hoyt Laboratory, Princeton University, 101 Hoyt Laboratory, William Street, Princeton, NJ 08544 USA; 2 Department of Chemical Engineering, University of Vigo, 36310 Vigo, Spain; 3 Department of Biotechnology Engineering, ORT Braude College, Karmiel, Israel; 4 Department of Chemical Engineering, Technion-Israel Institute of Technology, Haifa, Israel; 5 The Andlinger Center for Energy and the Environment, Princeton University, Princeton, NJ USA; 6 Department of Molecular Biology, Princeton University, Princeton, NJ 08544 USA; 7 Princeton Environmental Institute, Princeton University, Princeton, NJ 08544 USA

**Keywords:** Cellulose, Biofuels, Emulsions, Ethanol, Isobutanol, SSF, Thermotolerant, Optogenetics, Biomass pretreatment, *Saccharomyces cerevisiae*, *Ogataea polymorpha*, *Kluyveromyces marxianus*

## Abstract

**Background:**

Future expansion of corn-derived ethanol raises concerns of sustainability and competition with the food industry. Therefore, cellulosic biofuels derived from agricultural waste and dedicated energy crops are necessary. To date, slow and incomplete saccharification as well as high enzyme costs have hindered the economic viability of cellulosic biofuels, and while approaches like simultaneous saccharification and fermentation (SSF) and the use of thermotolerant microorganisms can enhance production, further improvements are needed. Cellulosic emulsions have been shown to enhance saccharification by increasing enzyme contact with cellulose fibers. In this study, we use these emulsions to develop an emulsified SSF (eSSF) process for rapid and efficient cellulosic biofuel production and make a direct three-way comparison of ethanol production between *S. cerevisiae*, *O. polymorpha*, and *K. marxianus* in glucose and cellulosic media at different temperatures.

**Results:**

In this work, we show that cellulosic emulsions hydrolyze rapidly at temperatures tolerable to yeast, reaching up to 40-fold higher conversion in the first hour compared to microcrystalline cellulose (MCC). To evaluate suitable conditions for the eSSF process, we explored the upper temperature limits for the thermotolerant yeasts *Kluyveromyces marxianus* and *Ogataea polymorpha*, as well as *Saccharomyces cerevisiae*, and observed robust fermentation at up to 46, 50, and 42 °C for each yeast, respectively. We show that the eSSF process reaches high ethanol titers in short processing times, and produces close to theoretical yields at temperatures as low as 30 °C. Finally, we demonstrate the transferability of the eSSF technology to other products by producing the advanced biofuel isobutanol in a light-controlled eSSF using optogenetic regulators, resulting in up to fourfold higher titers relative to MCC SSF.

**Conclusions:**

The eSSF process addresses the main challenges of cellulosic biofuel production by increasing saccharification rate at temperatures tolerable to yeast. The rapid hydrolysis of these emulsions at low temperatures permits fermentation using non-thermotolerant yeasts, short processing times, low enzyme loads, and makes it possible to extend the process to chemicals other than ethanol, such as isobutanol. This transferability establishes the eSSF process as a platform for the sustainable production of biofuels and chemicals as a whole.

**Supplementary Information:**

The online version contains supplementary material available at 10.1186/s13068-021-02008-7.

## Background

While traditional corn-derived ethanol has helped launch a sizable biofuel industry to combat greenhouse gas emissions in the transportation industry [1], challenges associated with expanding production from corn have made the utilization of more abundant feedstocks essential. These challenges largely stem from potential competition with the food industry as production expands [2] as well as the environmental impact on soil quality and prairie ecosystems as corn production increases [3]. Lignocellulosic biomass is an attractive alternative feedstock to address these problems, as cellulose is the most abundant organic polymer found in nature, which can be obtained from agricultural waste and energy crops grown on land not suitable for food production [4–6]. Because of these advantages, shifting biofuel processing from corn starch to cellulose opens the opportunity to expand production and replace a larger portion of fossil fuels with renewable energy while avoiding the negative environmental and financial impacts of using corn. Furthermore, as cellulose can be obtained from a variety of plants and wastes, the shift to cellulosic biofuels would allow for more geographic flexibility in the locations of biofuel production sites by expanding the possibilities beyond corn-rich regions [7].

Despite the necessity of cellulose for expanding sustainable biofuel production, its processing is more extensive than traditional feedstocks as biomass must first be deconstructed and saccharified into glucose before it can be converted into fuel. Several hydrolysis methods have been explored to break down cellulose into glucose, such as acid-catalyzed hydrolysis [8, 9], hydrolysis in subcritical and supercritical water [10], and enzymatic hydrolysis [11, 12]; however, only enzymatic hydrolysis prevents the high energy costs of elevated temperatures and the formation of toxic byproducts that inhibit the subsequent microbial fermentation. While enzymatic hydrolysis avoids these pitfalls of other methods, this approach suffers from slow and incomplete saccharification due to the tightly-packed crystalline structure of cellulose and feedback inhibition of the enzymes [6, 13, 14], which results in high enzyme costs and long processing times [15]. Because of this, there is much need for novel ways to improve enzymatic hydrolysis rates and conversion in cellulosic biofuel processes.

An effective approach to improve saccharification kinetics involves combining the hydrolysis and microbial fermentation into one step using a simultaneous saccharification and fermentation (SSF) process [16–20]. As the fermentation prevents accumulation of glucose in the culture, this approach avoids the effect of feedback inhibition, and thus increases the rate and extent of saccharification. While this approach is known to be more effective than separate hydrolysis and fermentation [21–23], the range of conditions at which the process can be performed is quite limited, as the hydrolysis conditions must be also compatible with yeast growth. Unfortunately, as the crystalline structure of cellulose cannot be quickly hydrolyzed at the low temperatures survivable for *Saccharomyces cerevisiae*, which is the yeast most often used for sustainable fuel and chemical production [24, 25], further improvements to the rate and extent of saccharification are still necessary [6, 26].

As the saccharification rate increases at elevated temperatures, thermotolerant yeasts which can survive and ferment at higher temperatures than *S. cerevisiae* have been identified as promising candidates for SSF processes [19, 27, 28]. Two thermotolerant yeasts that are well-suited for this application include *Kluyveromyces marxianus* [29–31] and *Ogataea* (*Hansenula*) *polymorpha* [32, 33], both of which can naturally ferment glucose into ethanol at high yields. These yeasts broaden the range of possible temperatures for the SSF process, as they can both ferment at up to 50 °C [29, 30, 33, 34], whereas the optimal temperature for *Saccharomyces cerevisiae* is only 30 °C [35, 36]. While both of these thermotolerant yeasts have been previously studied for their use in biofuel production, there is yet to be a three-way direct comparison of ethanol production between *S. cerevisiae*, *O. polymorpha*, and *K. marxianus*, and the use of *O. polymorpha* in an SSF process has not been reported. Moreover, there is much opportunity to improve thermotolerant SSF processes further using them in conjunction with other technologies that enhance saccharification.

Several pretreatment methods have been developed to deconstruct biomass prior to hydrolysis, including some using acids [37–40], ionic liquids [41–43] or alkaline solutions [9, 44]. However, giving enzymes full access to the cellulose polymer in biomass remains a challenge, limiting hydrolysis rates and conversion [6, 26]. A novel approach to enhance saccharification involves making emulsions from cellulose to further expand the exposed surface area [45]. These emulsions exploit the amphipathic nature of cellulose to stably coat oil microdroplets, making cellulose act as an emulsifying agent between the oil and water [46, 47]. This increases the surface area of cellulose exposed to cellulases, which enhances the rate of hydrolysis by as much as four-fold relative to rates of microcrystalline cellulose hydrolysis at 50 °C [45]. In addition, these oil-in-water emulsions open opportunities to develop one-pot processes in which the type of oil used is specifically selected to extract the product of interest, thus allowing saccharification, fermentation, and product separation to occur simultaneously. Saccharification gradually releases the oil microdroplets allowing them to coalesce into a separate phase; thus, using the oil to simultaneously extract the product would reduce product toxicity to microbial factories and facilitate downstream processing. Despite the promising advantages of cellulosic emulsions, their use in biomanufacturing, including SSF for biofuel production has not been demonstrated.

In this study, we combine SSF processing, conventional and thermotolerant yeasts, and cellulosic emulsions to achieve high ethanol yields in short processing times. We demonstrate that cellulosic emulsions enhance saccharification kinetics, leading to improved ethanol yields and productivities. In addition, we show the feasibility of using *O. polymorpha* in SSF processes, and present a three-way comparison of ethanol production between *S. cerevisiae*, *O. polymorpha*, and *K. marxianus* in glucose and cellulosic media. Furthermore, we extend the applications of the emulsified SSF (eSSF) process to produce the advanced biofuel isobutanol, which is, to our knowledge, the first report of cellulosic isobutanol production in yeast. This establishes the eSSF technology as a flexible platform for enhanced production of ethanol and other chemicals from cellulose.

## Results

### Enzymatic hydrolysis using a cellulose emulsion and microcrystalline cellulose

**Fig. 1:**
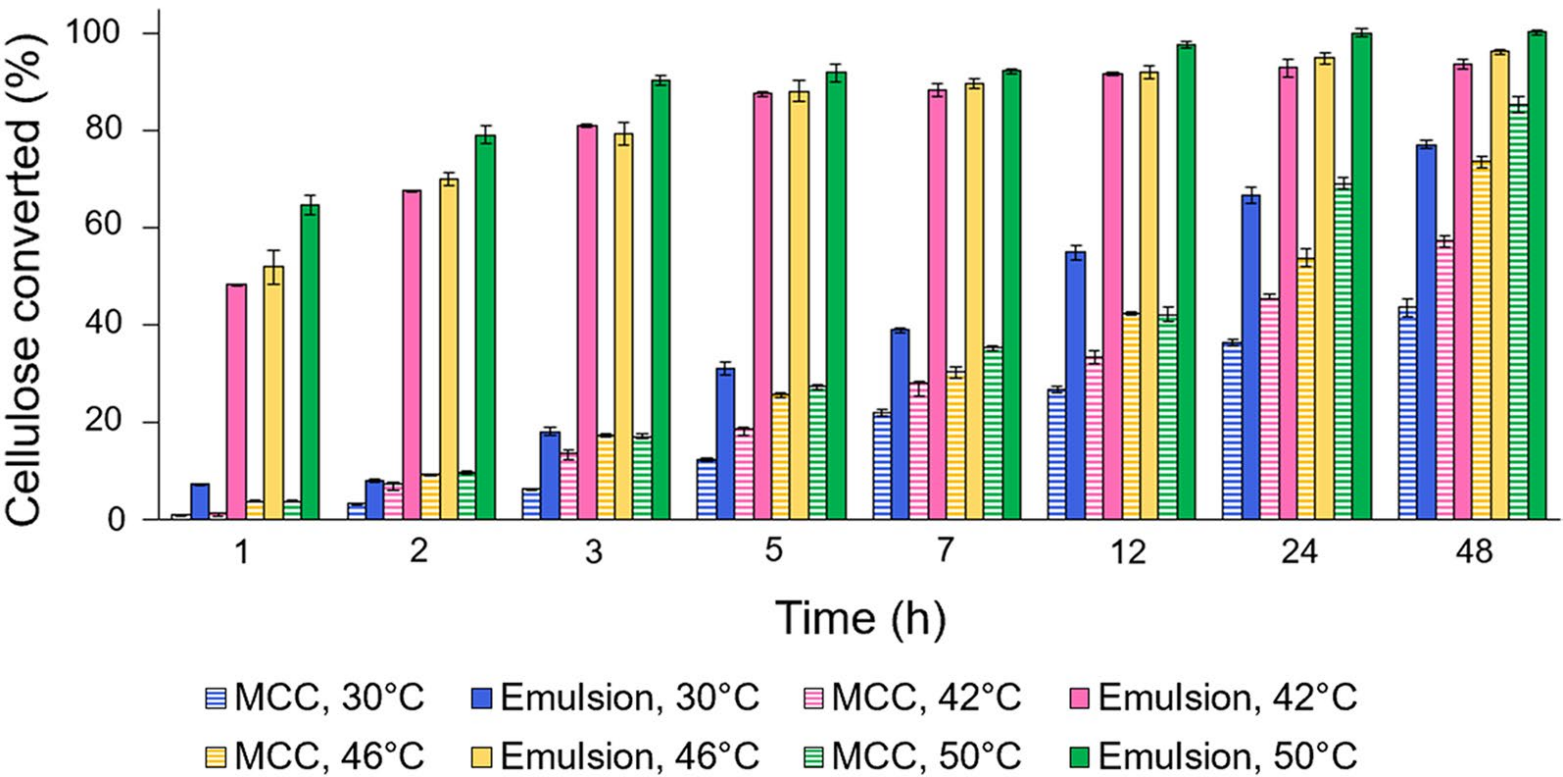
Comparison of cellulosic emulsion and microcrystalline cellulose hydrolysis kinetics. The conversion of cellulose was compared using a 0.6% cellulose emulsion and microcrystalline cellulose (MCC) at four temperatures and an enzyme load of 53 FPU/g substrate. Data is shown as the mean values and error bars represent the standard deviation of three replicates

Cellulose emulsions have been shown to hydrolyze faster and more completely than microcrystalline cellulose (MCC) at 50 °C [45], but since most yeasts do not tolerate temperatures as high as 50 °C, we sought to identify the best saccharification conditions at temperatures more suitable for an SSF process. To find the optimal conditions for hydrolysis, we performed comparative experiments using cellulosic emulsions and MCC samples at temperatures ranging from 30 to 50 °C, and at four cellulase concentrations (Fig. 1, Additional file 1: Figure S1). At all enzyme loads (with the possible exception of the lowest 7 FPU/g cellulose) and all temperatures examined, the emulsions hydrolyze faster and achieve higher extents of conversion than MCC samples of equal mass. This advantage is more pronounced at early stages of hydrolysis, reaching as much as 40-fold higher conversion of the emulsion at 42 °C, and 18-fold higher at 50 °C in the first hour (and 53 FPU/g substrate) than achieved with MCC under the same conditions (Fig. 1). While the optimal temperature for the cellulase cocktail we used is between 50 and 65 °C [48], the emulsions still reach nearly 80% conversion in 48 h at temperatures as low as 30 °C, which is the preferred temperature for non-thermotolerant yeasts like *Saccharomyces cerevisiae* [35]. The hydrolysis rate improves substantially when the temperature is increased to 42 °C, at which 80% conversion of the emulsion is reached in only 3 h. At the highest temperature examined, 50 °C, nearly full conversion of the emulsion is achieved within 12 h, while the MCC remains incompletely hydrolyzed even after 2 days. These results show that saccharification of the emulsions is faster and more efficient compared to MCC at temperatures that are significantly lower than the optimal for cellulase activity but suitable for yeast fermentation.

**Fig. 2:**
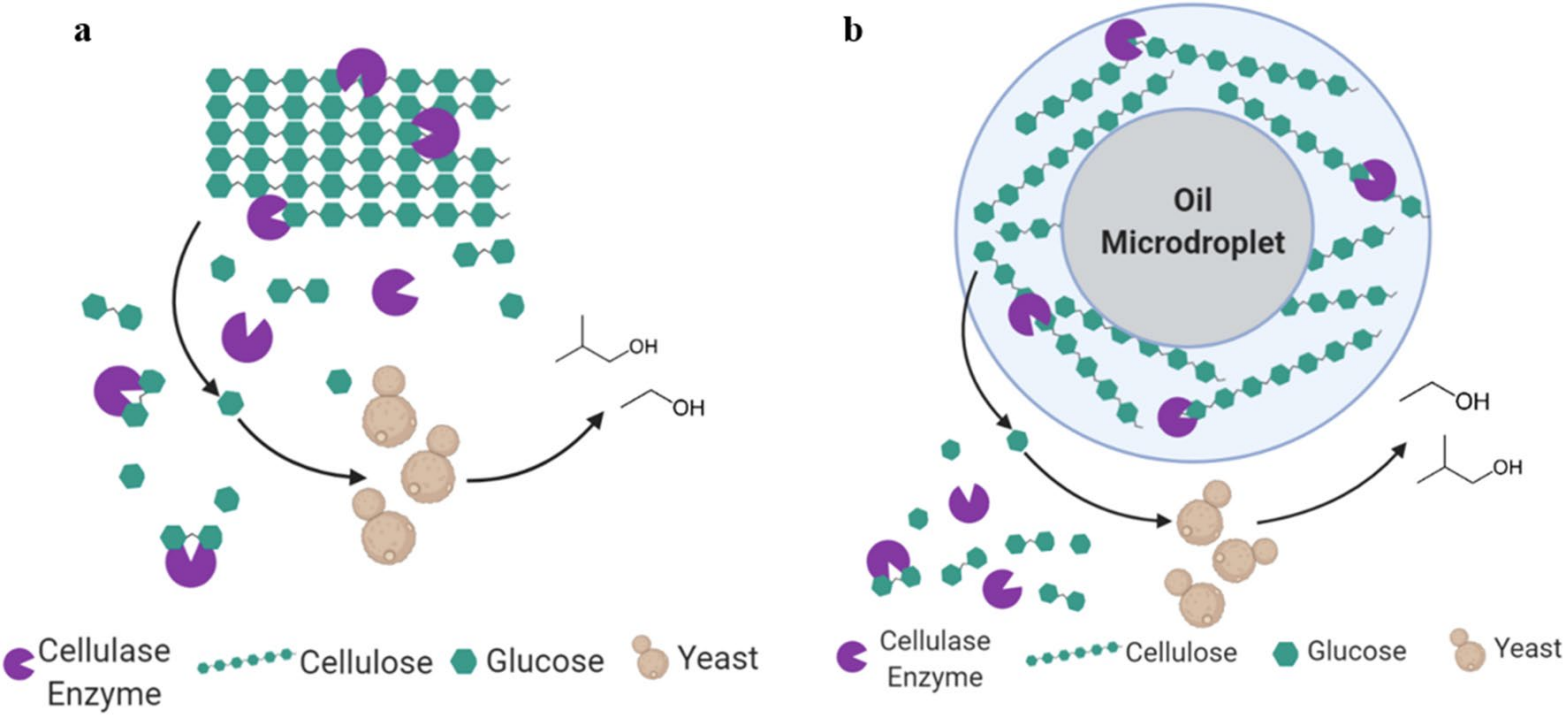
Simultaneous saccharification and fermentation (SSF) process schematic for cellulosic biofuel production. In both the **a** microcrystalline cellulose SSF (mcSSF) and **b** emulsified SSF (eSSF) processes, cellulase enzymes degrade cellulose into glucose, which is simultaneously metabolized by yeast into ethanol (or other chemicals). **a** In the mcSSF process, the untreated cellulose maintains a microcrystalline structure, which is more difficult for enzymes to hydrolyze. **b** In the eSSF process, the cellulose fibers coat the surface of oily droplets in the emulsion, providing better access to enzymes [45], and thus easier hydrolysis

While these results do indicate superior saccharification of the emulsion compared to MCC, the hydrolysis rates decrease as the reaction proceeds due to enzyme inhibition by the accumulating glucose [12]. In an SSF process in which yeast is grown simultaneously during hydrolysis (Fig. 2a), this inhibition is prevented as the glucose is consumed to produce desired chemicals as it is released [21, 22]. Because hydrolyzing cellulose in emulsions as opposed to microcrystals is advantageous at all temperatures tested between 30 and 50 °C (Fig. 1), the benefits of SSF and the emulsions can be combined within this full range of temperatures. Performing an emulsified simultaneous saccharification and fermentation (eSSF) process (Fig. 2b) using thermotolerant yeasts would take advantage of the higher cellulase activities at temperatures at the upper end of this range (42–50 °C). The improved kinetics of the emulsions also raises the opportunity to carry out the eSSF process at lower temperatures (30–42 °C) that are permissible for *S. cerevisiae*. Ultimately, using the emulsions in an eSSF process not only holds the practical advantages of condensing the saccharification and fermentation into one step, but also the potential to improve the hydrolysis kinetics even further than the rates measured in enzymatic tests (Fig. 1) by removing glucose inhibition of cellulases.

### Production of ethanol using thermotolerant yeasts

**Fig. 3:**
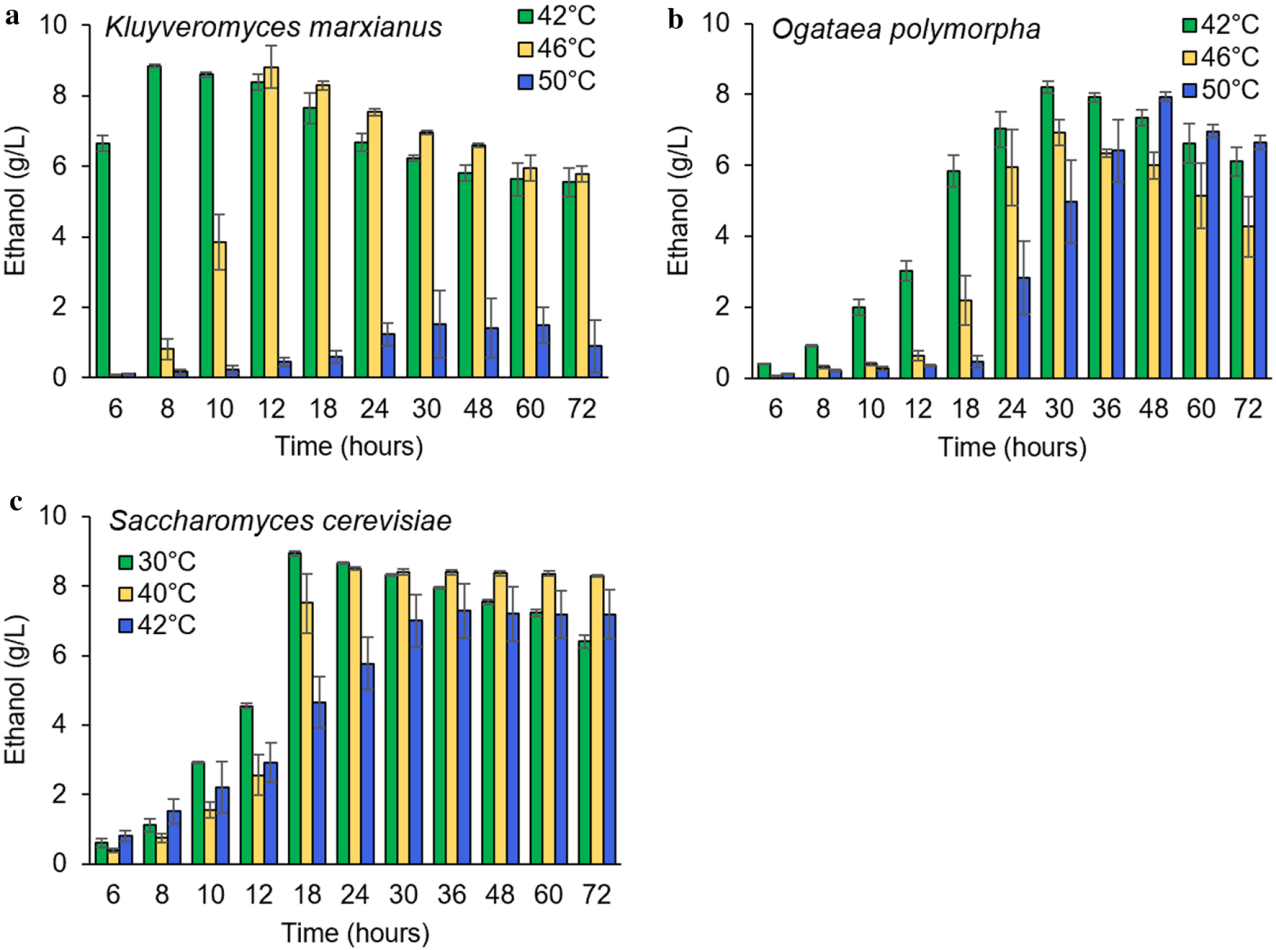
Three-way comparison of ethanol production from glucose by thermotolerant yeasts and *S. cerevisiae*. Ethanol production throughout time is shown for **a** *K. marxianus*, **b** *O. polymorpha,* and **c** *S. cerevisiae*, with 2% glucose provided as the carbon source. All data is shown as mean values and error bars represent the standard deviation of three independent replicates exposed to the same conditions

To choose an operating temperature for the SSF process, one needs to consider that cellulases are more active at higher temperatures, but also that there is a maximum temperature at which yeast can grow. To explore the upper temperature limit for fermentation, we first examined the ability of the thermotolerant yeasts *Kluyveromyces marxianus* and *Ogataea polymorpha*, as well as the common industrial yeast *Saccharomyces cerevisiae*, to ferment glucose into ethanol at different temperatures. The two thermotolerant yeasts, *K. marxianus* and *O. polymorpha*, both ferment well at elevated temperatures far beyond the 30 °C typically used for *S. cerevisiae* cultures (Fig. 3a, b). Of these yeasts, *K. marxianus* at 42 °C displays the highest ethanol titers and productivities compared to any other strain and temperature examined. While *K. marxianus* performs best at 42 °C, it is still able to produce high ethanol titers at temperatures up to 46 °C. Extending the benefits of thermotolerant yeasts even further, *O. polymorpha* maintains the unique advantage of being the only yeast that can grow and produce ethanol well at temperatures of up to 50 °C. For all fermentations, ethanol titers reach their maximum values as the glucose is fully consumed (Additional file 1: Figure S2). From that point on, ethanol concentrations steadily decrease (as long as fermentations are carried out under aerated conditions), suggesting there is a diauxic shift in which yeast begins to consume the ethanol. Aiming to operate SSF processes at temperatures as high as possible to favor cellulase activity, but still allow yeast to produce substantial amounts of ethanol, we chose to use 46 °C and 50 °C for *K. marxianus* and *O. polymorpha*, respectively.

We also explored the maximum permissible temperature for *Saccharomyces cerevisiae,* as it is widely used in the bioethanol industry and can be more readily engineered to synthesize many other valuable products [24]. We found that *S. cerevisiae* is able to ferment at up to 42 °C, which agrees with studies claiming this yeast is viable at temperatures of up to 45 °C [36] (Fig. 3c, Additional file 1: Figure S2c). Fermentations with this yeast achieve the highest ethanol titers and fastest production rate at 30 °C, reaching 8.9 g/L (87% of the overall theoretical yield) in 18 h. Though *S. cerevisiae* is able to ferment at 42 °C, which is better for cellulase activity than 30 °C, the yeast is not as productive at this temperature and achieves only 7.3 g/L ethanol (71% of the overall theoretical yield). As expected, and like the thermotolerant yeasts, *S. cerevisiae* undergoes a diauxic shift in aerated fermentations after glucose is fully consumed to assimilate the ethanol (Fig. 3c). This shift only occurs at 30 °C (Additional file 1: Figure S2c), which can be explained by the detrimental effect of high temperatures on respiration and mitochondrial activity previously reported [49–51]). Based on these results, 30 °C and 42 °C both offer potential advantages for SSF with *S. cerevisiae*: high ethanol titers and robust cell growth at 30 °C (Fig. 3c, Additional file 1: Figure S2c) and improved hydrolysis rates at 42 °C (Fig. 1).

### Simultaneous saccharification and fermentation process for ethanol production

**Fig. 4:**
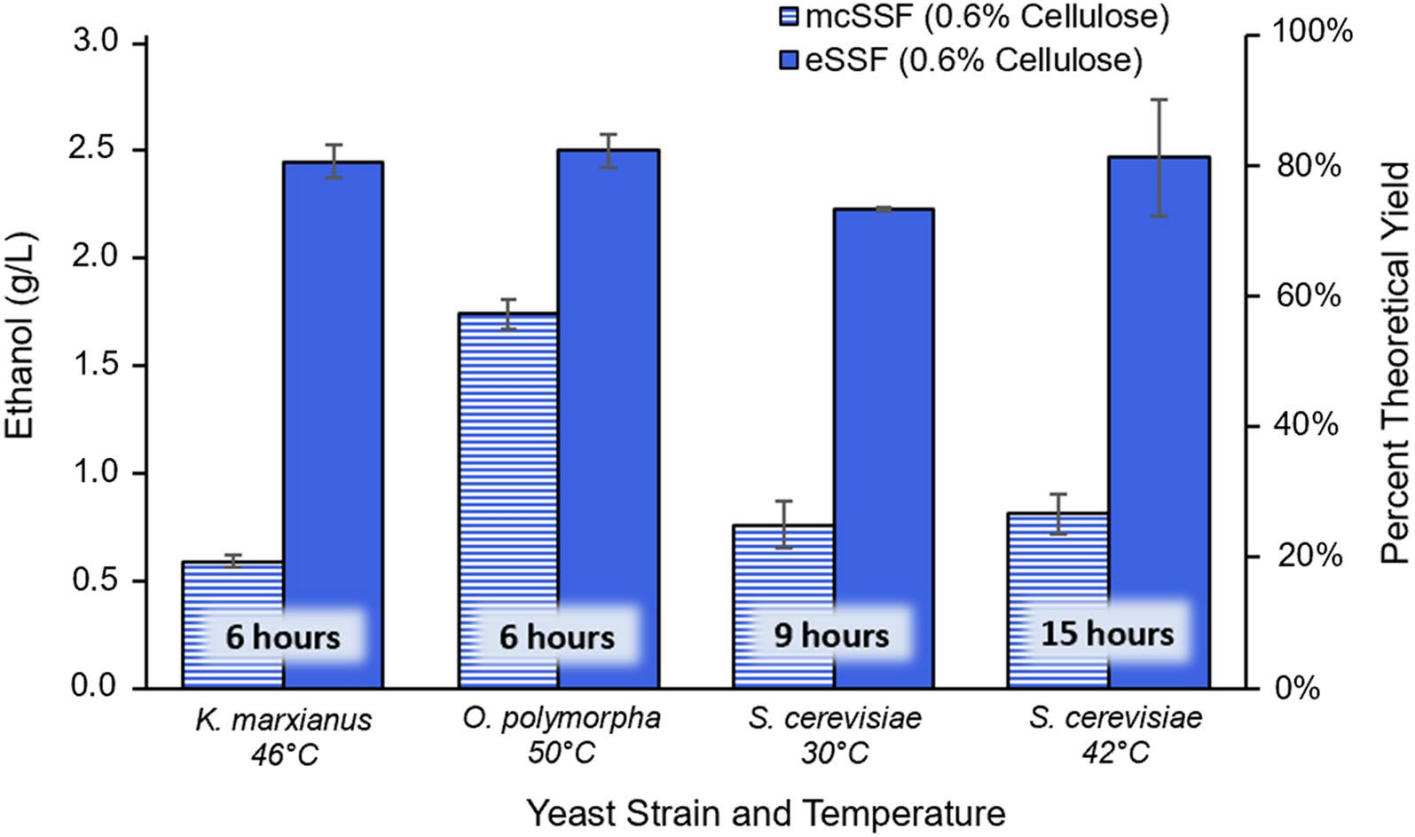
Ethanol production using eSSF or mcSSF processes. Ethanol titers obtained from the mcSSF and eSSF processes show that higher ethanol production is consistently achieved from cellulosic emulsions than from microcrystalline cellulose. The highest ethanol concentrations reached with *K. marxianus*, *O. polymorpha*, and *S. cerevisiae* using a 0.6% cellulose emulsion (eSSF) or microcrystalline cellulose (mcSSF) are depicted. The times when maximum ethanol titer are reached for each yeast are shown. An enzyme load of 53 FPU/g cellulose was used for all yeasts, and data is shown as mean values and error bars represent the standard deviation of three independent replicates

With suitable temperatures identified to balance the kinetic and metabolic optima for saccharification and fermentation, we compared SSF processes using MCC (mcSSF) or emulsified cellulose (eSSF) to produce ethanol using the three yeast species tested above. Both mcSSF and eSSF processes were carried out with a cellulase load of 53 FPU/g cellulose, using a 0.6% cellulose emulsion for eSSF processes, equivalent to 0.59 g/L of glucose in the media (see “Methods”), and monitoring ethanol concentrations throughout 48 h of fermentation (Additional file 1: Figure S3). For all three yeasts, the eSSF process produces more ethanol than the mcSSF at the same cellulose concentration and enzyme load (Fig. 4). In the cases of *K. marxianus* and *S. cerevisiae*, the emulsion is advantageous over the microcrystalline cellulose by at least ~ 1.4-fold and up to 4.2-fold (Fig. 4), with ethanol yields from eSSF ranging between 73 and 81% of the theoretical value, compared to an average of about 24% for the mcSSF (Additional file 1: Figure S3). The advantage of eSSF is still observed with *O. polymorpha* fermentations but shows a lower enhancement of approximately 1.5-fold higher titers relative to mcSSF, probably because the higher temperature (50 °C), enhances cellulase activity and thus hydrolysis during mcSSF (Fig. 4). This suggests that the benefits of eSSF are mainly borne at temperatures between 30 and 46 °C, which encompasses the most common operating temperatures of yeast fermentations. In addition to the advantages of eSSF over mcSSF, these results also confirm that eSSF achieves higher rates and extents of saccharification over separate enzymatic hydrolysis, as simultaneous fermentation prevents feedback inhibition of the cellulases. This advantage can be observed for all yeasts, with the possible exception of *S. cerevisiae* at 42 °C, by comparing the percent conversion throughout time of separate enzymatic hydrolyses (Fig. 1) with the minimal conversion observed in the eSSF processes (see “Methods”) (Additional file 1: Figure S4). In the case of *S. cerevisiae* at 42 °C, the high temperature slows the fermentation rate, leading to underestimation of the cellulose hydrolysis rate, as this was indirectly measured from the rate of ethanol production (see methods). Ultimately, eSSF is superior to both mcSSF and separate hydrolysis by achieving faster saccharification and higher ethanol titers with all three yeast species.

While the eSSF process outperforms mcSSF at all conditions tested, especially at temperatures below 50 °C, fermentations at elevated temperatures with thermotolerant yeasts are especially productive compared to lower temperature fermentations. Whereas *S. cerevisiae* requires between 9 and 15 h (at 30 °C and 42 °C, respectively) to reach its maximum ethanol titers, this time is decreased by at least a third when using *K. marxianus* or *O. polymorpha* (at 46 °C and 50 °C, respectively), which fully ferment within 6 h (Fig. 4, Additional file 1: Figure S3). This shorter fermentation time is due to increased hydrolysis rate at high temperatures, further enhanced by the continuous glucose consumption of the eSSF, which prevents cellulase inhibition (Additional file 1: Figure S4). In addition to completing the fermentation in less time than *S. cerevisiae*, the titers obtained with the thermotolerant yeasts are equivalent or slightly higher than those obtained with *S. cerevisiae*, which highlights that the shortened fermentation time and enhanced productivity does not come at a cost to the overall yield.

### Improving eSSF for *S. cerevisiae* fermentations

Contemplating the greater goal of using eSSF to produce cellulosic chemicals beyond ethanol, we focused our attention on *S. cerevisiae,* since it has the most engineering tools and strains available for the production of valuable chemicals compared to the other yeasts [24]. To boost eSSF production further with this yeast, we explored whether it was possible to raise titers by increasing the cellulose load of the emulsion from 0.6 to 2% by weight (equivalent to 20 g/L of glucose in the fermentation media). For this new emulsion, we used a hexadecane content of 0.6%, which was the same as the previous emulsion.

**Fig. 5:**
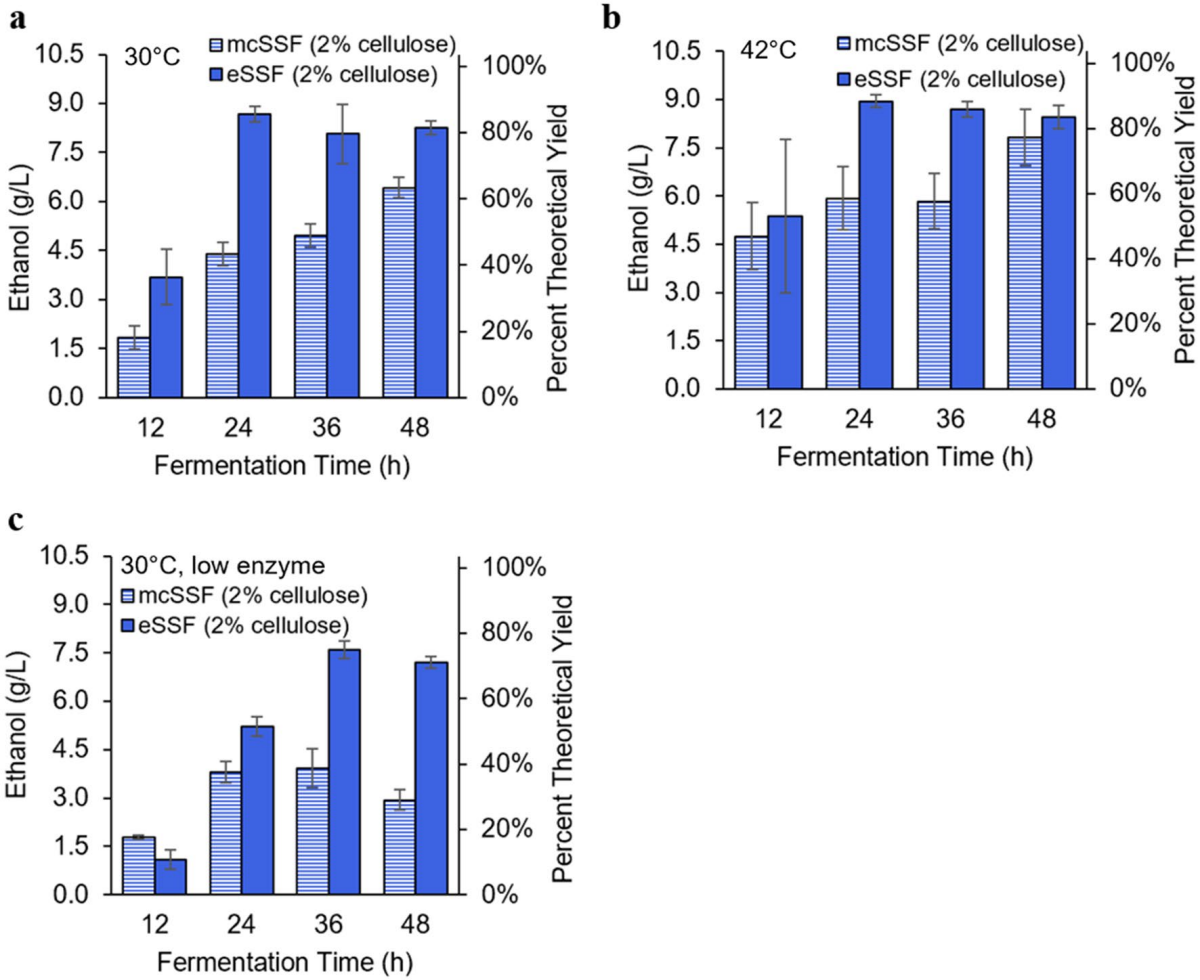
Ethanol production in *S. cerevisiae* using an increased cellulose load in the eSSF or mcSSF processes. Ethanol titers and percent theoretical yield obtained from *S. cerevisiae* in eSSF or mcSSF processes using a 2% cellulose content at both **a** 30 °C and **b** 42 °C with an enzyme load of 53 FPU/g cellulose. At this enzyme load, the eSSF process reaches at least 86% of the theoretical yield within 24 h at both temperatures. The eSSF and mcSSF processes are also compared at a lower enzyme load of 26 FPU/g cellulose at 30 °C (**c**), with the eSSF process exceeding the mcSSF titers by a factor of two after 36 h. Data is shown as mean values and error bars represent the standard deviation of three independent replicates

As expected, increasing the cellulose content in the emulsion boosts ethanol titers, and outperforms equivalent increases in microcrystalline cellulose in terms of both yield and fermentation time (Fig. 5**)**. Emulsions with 2% cellulose approach full hydrolysis in eSSF processes within 24 h at both 30 °C and 42 °C, while mcSSF processes using 2% MCC do not reach full hydrolysis even in 48 h at either temperature (Additional file 1: Figure S5). In addition to hydrolyzing faster, cellulosic emulsions also achieve higher ethanol titers and productivities than fermentations carried out with MCC regardless of temperature, reaching nearly 90% theoretical yield at both 30 °C and 42 °C within 1 day (Fig. 5 a, b). To further demonstrate the potential of cellulosic emulsions to enhance productivity in more challenging conditions, we cut the enzyme load by a factor of two (from 53 FPU/g cellulose to 26 FPU/g cellulose) while fermenting at 30 °C (Fig. 5c). Even at this lower enzyme load, the eSSF process still reaches nearly 80% theoretical yield within 36 h, which is nearly double the output of the mcSSF process in the same period of time.

Furthermore, to reduce yeast ethanol consumption, we conducted the 2% cellulose fermentations without aeration (Fig. 5). These results show that the eSSF process increases ethanol titers and productivities relative to microcrystalline cellulose at concentrations equivalent to at least 2% glucose, which raises the possibility of using this method to produce other valuable chemicals from cellulose with engineered strains of *S. cerevisiae*.

### Isobutanol production using an optogenetically controlled eSSF process

**Fig. 6:**
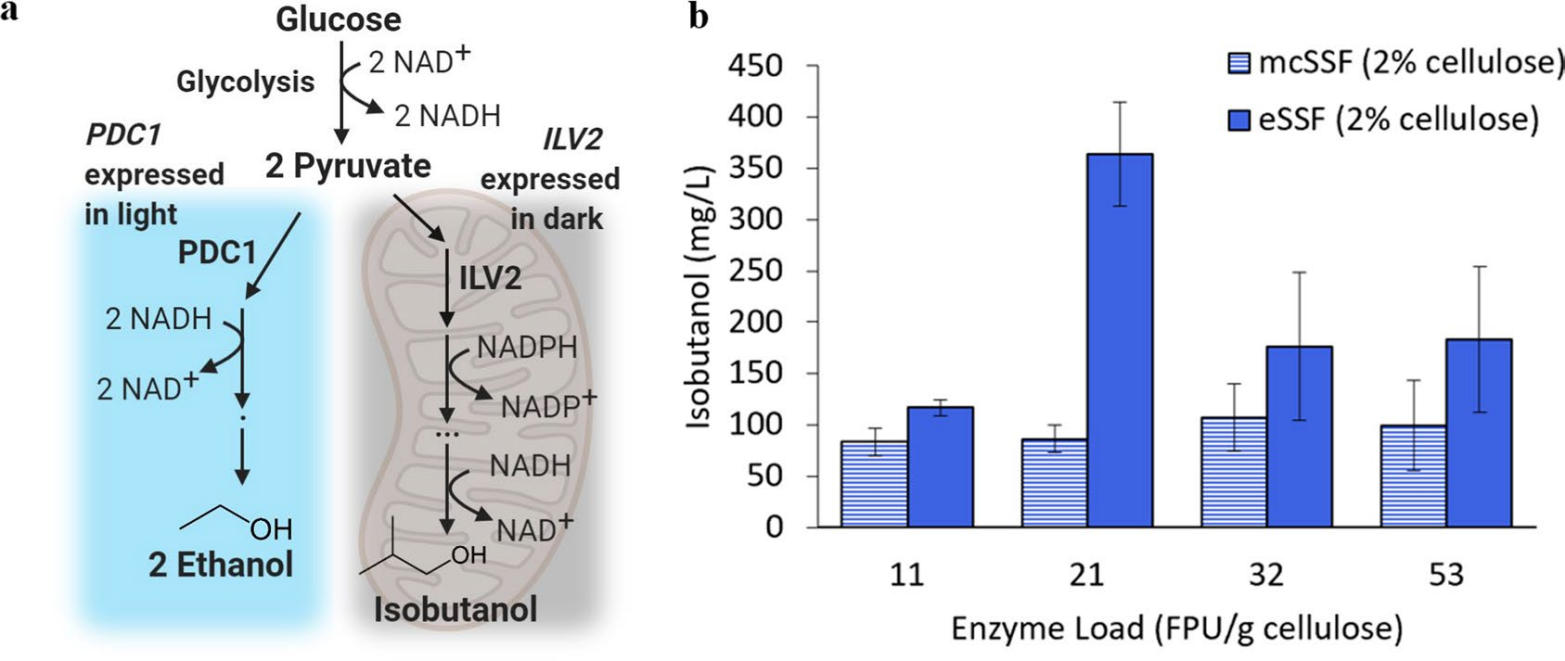
Cellulosic isobutanol production using an optogenetically controlled eSSF process. **a** Schematic of the optogenetically controlled isobutanol and ethanol biosynthetic pathways, which compete for pyruvate from glycolysis. The enzymes which catalyse the first steps of each of these pathways are placed under optogenetic control (see “Methods”), with *PDC1* of the ethanol pathway expressed in the light, and *ILV2* of the isobutanol pathway expressed in the dark. The isobutanol pathway is localized to the mitochondria, whereas ethanol biosynthesis occurs in the cytosol. **b** Isobutanol titers recorded in eSSF and mcSSF experiments at 30 °C, using the optogenetic *S. cerevisiae* strain YEZ546-2 and different enzyme loads. All tests used either a 2% cellulose emulsion or 2% microcrystalline cellulose mixture and were switched from the light-driven growth phase to the darkness-induced production phase at the same cell density. Mean values are shown, and error bars represent the standard deviation of three independent replicates

To demonstrate the potential of extending the benefits of eSSF to the production of other chemicals, we used it with engineered *S. cerevisiae* strains to produce isobutanol, an advanced biofuel that can be used as a drop-in gasoline substitute or upgraded to jet fuel [52, 53]. Biosynthesis of isobutanol in yeast is challenged by the strong competition with ethanol biosynthesis, a pathway that cannot be easily deleted genetically due to its essential role for growth on glucose [54, 55]. We have previously shown that this challenge can be overcome by dynamically controlling the ethanol and the mitochondrial isobutanol biosynthetic pathways with light using optogenetic circuits [56, 57]. The two pathways compete for pyruvate, metabolized by either pyruvate decarboxylases (encoded by *PDC1, PDC5* and *PDC6*) for ethanol production or by acetolactate synthase (encoded by *ILV2*) for isobutanol. Therefore, these enzymes can be used as optogenetic metabolic valves to dynamically direct flux towards either pathway (Fig. 6a). By controlling *PDC1* (in a strain with the three native *PDC* genes knocked out) using a light-activated circuit (OptoEXP) [57], and *ILV2* with a dark-activated circuit (OptoINVRT7) [56], the engineered yeast can grow only in the light (producing ethanol), and then direct its metabolic flux towards isobutanol production in the dark (Fig. 6a). This approach has been shown to improve isobutanol titers by allowing biomass to build up before repressing an essential competing pathway and subjecting the cells to the metabolic burden of production [56, 57].

Using this light-responsive engineered strain, we compared isobutanol production from 2% cellulose in eSSF and mcSSF processes (see “Methods”). Testing a range of cellulase enzyme loads, we found that, similar to the ethanol process, eSSF enhances cellulosic isobutanol production compared to mcSSF at equivalent cellulose and enzyme loads (Fig. 6b). Furthermore, we found that the optimal enzyme load for isobutanol production is lower than what achieves the highest rate of hydrolysis. The increase in isobutanol production at moderate enzyme loads could potentially be attributed to the fact that faster hydrolysis can lead to a higher concentration of glucose in the medium, which can suppress mitochondrial activity (where the isobutanol pathway is located) [58, 59]. At the optimal load of 21 FPU/g cellulose, the emulsion fully hydrolyzes and produces isobutanol titers up to 364 mg/L, which is over fourfold higher than the mcSSF process. Unlike the emulsion, the microcrystalline cellulose does not fully hydrolyze within 48 h, indicating that the low enzyme loads compatible with the emulsions are not enough for complete saccharification of the microcrystalline cellulose. Overall, these results indicate a substantial improvement in cellulosic isobutanol production when using the emulsions compared to microcrystalline cellulose.

## Discussion

In this study, we report both a three-way comparison of ethanol production between *S. cerevisiae*, *O. polymorpha*, and *K. marxianus* at different temperatures as well as the effect that the eSSF technology has on productivity. We draw comparisons between these three species both in glucose and cellulosic media at a range of temperatures from 30 to 50 °C, which show *O. polymorpha* to have the greatest thermotolerance among the species examined. This constitutes, to our knowledge, the first side-by-side comparison of ethanol production between these three species and offers insights into their relative productivities, yields, and temperature limitations. While temperature limitations will be strain-dependent in any future application, these insights facilitated selection of appropriate temperature conditions for our strains as we pursued the development of the eSSF process.

This study also addresses three key challenges of cellulosic biofuel production: incomplete cellulose conversion, slow hydrolysis rate, and high enzyme costs, which stem from the tightly-packed crystalline structure of cellulose and feedback inhibition of the cellulase enzymes [14, 15]. Cellulase hydrolysis is favored at elevated temperatures and optimal activity of commercial cellulase cocktails is between 50 and 65 °C [19, 48]. Combining the saccharification and fermentation of cellulose into one step using an SSF process can prevent feedback inhibition, but for these processes to be effective, the saccharification must be performed at conditions that are permissible for yeast. In this study, we exploit the improved hydrolysis kinetics of cellulosic emulsions [45], to enable eSSF processes at temperatures that are both effective for saccharification and tolerable for yeast fermentation. Even without simultaneous fermentation preventing feedback inhibition, these emulsions can be almost fully hydrolyzed (90–100%) at temperatures suitable for thermotolerant yeasts (42–50 °C) in 3–12 h, and reach ~ 75% conversion at temperatures as low as 30 °C in 2 days (Fig. 1). However, saccharification kinetics are further enhanced to nearly full conversion to ethanol within 6 h in eSSF processes at 46–50 °C using the thermotolerant yeasts *K. marxianus* and *O. polymorpha* (at 53 FPU/g cellulose of enzyme)*.* These elevated temperatures slow fermentation rates (Additional file 1: Figure S2), which could lead to an underestimation of the cellulose hydrolysis rate making this a conservative comparison between the eSSF and separate enzymatic hydrolysis experiments.

The yields and fermentation times achieved using this eSSF technology improve upon previous literature using different pretreatments. When using *S. cerevisiae* at a similar enzyme load, the eSSF process reaches a similar percent theoretical yield of ethanol in half the time (86% in 24 h, Fig. 5a, b) compared to SSF processes using phosphoric acid pretreatment (89% in 48 h) [60]. Our eSSF also outperforms SSF with alkaline pretreatment, even at a significantly lower temperature*,* reaching 75% theoretical yield in just 36 h at 30 °C (Fig. 5c) compared to 65% in 72 h at 38 °C [61]. The advantage of the eSSF technology is also apparent when using the thermotolerant yeast *K. marxianus*, reaching 81% of theoretical yield in 6 h compared to 58% in 72 h in SSF processes using ammonia fiber explosion pretreatment, although these two studies use substantially different cellulose loads, which complicates comparison [62].

The increase in saccharification rates using eSSF mimics the kinetic advantages of raising the temperature in cellulosic hydrolysis. The benefit of eSSF is especially dramatic at relatively low temperatures with the commonly used *S. cerevisiae*, which reaches nearly full conversion of cellulose to ethanol at 30 °C within a day (Additional file 1: Figure S5). In comparison, the mcSSF process fails to reach full conversion at temperatures as high as 42 °C even after 48 h. The kinetic similarities between using the emulsions and raising the temperature are also apparent from the enzymatic hydrolysis experiments (without simultaneous fermentation). These results show that nearly full hydrolysis is achieved with enzyme loads as low as 26 FPU/g cellulose (Additional file 1: Figure S1a), which does not occur for the MCC even at 50 °C, after 48 h, and an enzyme load twice as high (Fig. 1). Therefore, eSSF processes enhance hydrolysis at lower temperatures, reduce processing times, and lower enzyme loads. As enzyme cost is a major fraction of the overall cost of cellulosic ethanol production, comprising as much as 48% of the minimum ethanol selling price [55], these advantages open the possibility for eSSF to substantially improve the viability of cellulosic biofuels.

An unexpected finding was that eSSF and, to a lower extent, mcSSF enhance the viability and productivity of *S. cerevisiae* at 42 °C. Fermentations in glucose at this elevated temperature exhibit reduced growth and incomplete glucose consumption (Additional file 1: Figure S2c). In contrast, eSSF processes at this temperature reach nearly full cellulose consumption and conversion of the released glucose to ethanol (Fig. 5, Additional file 1: Figure S3d). In addition, the productivity at 42 °C is higher when using eSSF with 2% cellulose tan direct fermentation of 2% glucose, most evidently seen when comparing titers at 12 and 24 h (Figs. 3c, ). In fact, the productivity of eSSF processes with this yeast is higher at 42 °C than 30 °C, probably due to the enhanced rate of hydrolysis at higher temperatures (Fig. 5, Additional file 1: Figure S3c-d). This contrasts with our observations in glucose fermentations, where the productivity at 30 °C is the highest (Fig. 3c). We hypothesize that this apparent thermotolerance enhancement is related to the rate of glycolysis, which is likely reduced in eSSF processes as glucose concentrations remain low throughout the fermentation (as glucose is consumed as soon as it is released by cellulose hydrolysis). This unexpected improvement in thermotolerance of *S. cerevisiae* in cellulosic eSSF processes, probably brought on by throttling metabolic rates, warrants further investigation.

Beyond the enhanced production of cellulosic ethanol with eSSF, the ability to perform the process at temperatures compatible with non-thermotolerant yeasts like *S. cerevisiae* allows for the benefits of eSSF to be extended to produce a wide range of chemicals other than ethanol. The ability to use *S. cerevisiae* is particularly valuable as this yeast has the largest genetic engineering toolbox available and has been engineered to produce the broadest range of renewable chemicals [24]. We demonstrate this with strains engineered to produce isobutanol, a next-generation biofuel with superior fuel properties compared to ethanol [63]. We found eSSF achieves significantly higher isobutanol titers compared to microcrystalline cellulose (Fig. 6b). This transferability establishes the eSSF process as a promising platform to produce cellulosic biofuels and chemicals beyond only ethanol.

As we transferred the eSSF process from ethanol to isobutanol production, we found that production is not necessarily optimized at the maximum saccharification rate. In fact, maximum isobutanol production occurs at an enzyme load of 21 FPU/g cellulose, which is 60% lower than the load used in most of our ethanol experiments (Fig. 6b). We hypothesize that a moderate enzyme load is optimal for isobutanol production because of the Crabtree effect of *S. cerevisiae*, which causes glucose to be preferentially converted to ethanol [64], and suppression of mitochondrial activity [58, 59, 65]. While the optogenetic controls of the *S. cerevisiae* strain in this study aim to reduce ethanol production in the dark, these controls do not completely inhibit ethanol production. Therefore, the lower glucose concentrations resulting from slightly slower hydrolysis could lessen the Crabtree effect and favor isobutanol production [66]. While moderate saccharification rates benefit isobutanol production, rather than the fastest hydrolysis achievable with eSSF, these emulsions remain advantageous for this application as they allow for even lower enzyme loads than microcrystalline cellulose. These results highlight the importance of optimizing the enzyme load in eSSF processes when producing cellulosic chemicals other than ethanol, as controlling hydrolysis rate can help reduce byproducts and costs.

While the emulsions used in this study hold significant advantages, there is still room for improvement. The emulsion composition and structure should be optimized by varying cellulose and oil concentrations, choices of oil, and microdroplet sizes to further enhance cellulase activity and saccharification kinetics at low temperatures. Increasing the cellulose load of emulsions is particularly important for the viability of this approach. Therefore, future work should especially focus on overcoming the technical challenges associated with preparing high-cellulose emulsions, such as their high viscosity. Moreover, preparing emulsions using oils with high extraction coefficients for isobutanol or other hydrophilic products [67] could raise the possibility of combining simultaneous saccharification and fermentation with product separation, as these compounds are sequestered into the oily droplets of the emulsion away from the aqueous culture, which would reduce their toxic effects on microbial cell factories. As the cellulose is consumed, the emulsions are destabilized and the oily droplets coalesce into a continuous separate phase, which would also assist with downstream processing and product purification. Emulsions containing both cellulose and hemicellulose, and development of eSSF processes using strains engineered to co-utilize glucose and xylose [68, 69] are also valuable areas of future research. Finally, as enzymes remain a major cost in cellulosic bioprocesses, research to further reduce the necessary enzyme loads will enhance the scalability of this technology. Exploring these improvements in the eSSF process could potentially lead to higher titers, shorter processing times, and lower production costs for cellulosic chemicals.

Ultimately, the eSSF process addresses several key technical challenges of cellulosic biofuel production: incomplete hydrolysis, slow saccharification rate, and high enzyme costs. The rapid hydrolysis of these emulsions at low temperatures permits fermentation using non-thermotolerant yeasts, the production of chemicals beyond ethanol, shorter processing times, and substantially lower enzyme loads compared to traditional cellulosic biofuel production processes. These advantages, combined with the low cost of reagents used to make these emulsions, establishes this technology as a valuable, practical, and economical tool for the sustainable production of cellulosic biofuels and chemicals, with much opportunity to build upon these results even further.

## Conclusions

In this study, we used cellulosic emulsions to increase saccharification efficiencies at low temperatures tolerable to both thermotolerant and non-thermotolerant yeasts. We show rapid enzymatic hydrolysis compared to microcrystalline cellulose at a wide range of enzyme loads and temperatures, with these saccharification kinetics improved even further in the eSSF process. In the eSSF process, close to theoretical cellulosic ethanol yields are reached within 1 day even at temperatures as low as 30 °C. This performance at low temperatures opens the possibility to use the eSSF process to sustainably produce other chemicals using *S. cerevisiae*, which we demonstrate through the successful production of isobutanol in an optogenetic eSSF process. Ultimately, this eSSF technology allows for efficient conversion of cellulose to biofuels using short process times and low enzyme loads, and sets a new flexible foundation for the production of cellulose-derived chemicals in general.

## Methods

### Preparation of cellulosic emulsions

To prepare the cellulosic emulsions, we first dried the microcrystalline cellulose powder (Avicel) in a vacuum oven at 60 °C and 0.26 kPa for at least 24 h. The powder was then dissolved in phosphoric acid by mixing 4 wt% microcrystalline cellulose and 6 wt% deionized water with phosphoric acid 85% (Bio-Lab Ltd, Israel) in a 0 °C cooling bath. The solution was mixed for 2 h at 500 rpm, until no crystalline particulates could be observed visually. This cellulose solution was then coagulated by adding deionized water. We continued to rinse the coagulated hydrogel with excess deionized water to remove phosphoric acid traces until electrical conductivity of the wash water was below 1 mS cm^−1^. After rinsing, water and sodium acetate buffer solution were added to the hydrogel in accordance with the desired cellulose concentrations (accounting for the hexadecane added in future steps) and a final buffer concentration of 25 mM. The hydrogel particles were then dispersed using an IKA® T-18 Ultra-Turrax® mechanical homogenizer (IKA Works Inc., Germany) at 18,000 rpm to reduce particle size, after which we determined the cellulose content gravimetrically.

From the dispersed hydrogels, we fabricated the cellulose/hexadecane emulsions in two stages. First, a pre-emulsion was obtained by mixing hexadecane (Merck Chemicals, Israel) with the aqueous cellulose hydrogel dispersion using a IKA® T-18 Ultra-Turrax® mechanical homogenizer (IKA Works Inc., USA) at 18,000 rpm for 5 min, and then this coarse emulsion was further emulsified using a microfluidizer (Model LM-20, Microfluidics, USA). Emulsification was done by circulating the sample in the air-driven microfluidizer through a 100 µm Z channel for 4 min. The working pressure of the liquid through the channel was about 140 MPa and temperature was maintained at approximately 50 °C using ice. We fabricated emulsions at two different cellulose/hexadecane compositions (%wt. cellulose/%wt. hexadecane), including 0.6/0.6 and 2/0.6.

### Enzymatic hydrolysis and enzyme activity assay

Experiments characterizing the enzymatic hydrolysis rate (in the absence of yeast) were performed on a 0.6% cellulose/0.6% hexadecane emulsion and a mixture containing 0.6 wt% microcrystalline cellulose (Avicel) and 25 mM sodium acetate buffer (pH = 4.8) in deionized water. The hydrolysis was performed in 50-mL falcon tubes containing 15 mL of either the emulsion or microcrystalline cellulose (MCC) solution inoculated with the Accellerase 1500 commercial enzyme cocktail (Genencor) at the specified enzyme loads (7, 13, 26, and 53 FPU/g cellulose). The samples were incubated at several temperatures (30, 42, 46, and 50 °C) with shaking at 200 rpm on an orbital shaker until harvesting at each of the designated timepoints (Fig. 1 and Additional file 1: Figure S1) (Innova 4000, New Brunswick). At each timepoint, 1 mL was sampled from each tube and heated to 100 °C for 10 min to inactivate the cellulases and prevent any further hydrolysis in the time between sampling and analysis. The cellulose in each sample was separated from the aqueous solution by centrifuging for 5 min at 3500* g* in a table-top centrifuge (Eppendorf 5424R). 90µL of the aqueous phase was harvested to perform the enzyme activity assay, which was carried out using the dinitrosalicylic acid (DNS) method [70]. To perform this method, the 90µL of aqueous sample was added to 1 ml DNS solution, heated to 100 °C for 10 min, and cooled to ambient temperature. The reduced sugar concentration was measured by colorimetry at 575 nm in an Eppendorf spectrophotometer (BioSpectrometer basic) and the recorded values were converted to grams reduced sugar per liter by also analyzing standard samples of known reduced sugar concentrations. We then related the concentration of reduced sugars to the initial quantity of cellulose to calculate the percent conversion of cellulose.

### Yeast strains and transformations

The thermotolerant yeasts *Kluyveromyces marxianus* (ATCC 26548) and *Ogataea polymorpha* (ATCC MYA-336) were acquired from the American Type Culture Collection (ATCC). The *Saccharomyces cerevisiae* strain used for ethanol production, MAy_6, was obtained by transforming SHy48 [71], a strain derived from CEN.PK2, with the EZ-L350 plasmid [57], which constitutively expresses GFP from the TEF1 promoter using *HIS3* as the marker. This incorporation of GFP was performed to relate fluorescence and cell density in the presence of cellulose (when optical density readings cannot be acquired); however, the results of these experiments are not included due to the instability of GFP at elevated temperatures, which limited the effectiveness of this approach. This transformation was performed using a standard lithium acetate protocol.

For the isobutanol tests, we used the *S. cerevisiae* YEZ546-2 strain, which is engineered with dynamic optogenetic controls to grow robustly in the light and produce isobutanol in the dark [56]. In this strain, the native *PDC1*, *PDC2*, and *PDC3* genes are deleted, which are essential for fermentation of glucose into ethanol. To allow for growth and ethanol production exclusively in the light, which reduces competition between the isobutanol and ethanol pathways in the dark production phase, an exogenous copy of *PDC1* is expressed under a light-activated circuit. This strain also contains the mitochondrial isobutanol pathway in a 2µ plasmid, with the first enzyme of this pathway, *ILV2*, expressed under a light-repressed circuit such that isobutanol production occurs only in the dark.

### Ethanol production from glucose using thermotolerant yeasts

We investigated the effect of temperature on ethanol titers and fermentation timescales using *K. marxianus* strain ATCC 26548, *O. polymorpha* strain ATCC MYA-336, and *S. cerevisiae* strain MAy_6. Each thermotolerant yeast was cultivated in specific media recommended for culturing conditions by ATCC, while synthetic dropout medium was used for MAy_6 (*S. cerevisiae*); all media were supplemented with 2% glucose. *K. marxianus* was cultivated in YMPD medium prepared with 3 g l^−1^ yeast extract, 3 g l^−1^ malt extract, and 5 g l^−1^ peptone. The second thermotolerant yeast, *O. polymorpha*, was grown in a YPD medium containing 10 g l^−1^ yeast extract, 20 g l^−1^ peptone, and 30 mg l^−1^ supplements of additional leucine and uracil. Finally, *S. cerevisiae* strain MAy_6 was grown in an SC dropout medium containing 3 g l^−1^ yeast nitrogen base without amino acids or ammonium sulfate, 10 g l^−1^ ammonium sulfate, 72 mg l^−1^ inositol, and 2 g l^−1^ amino acid mixture lacking uracil and histidine.

For the *K. marxianus* experiments, we examined ethanol production at 42, 46, and 50 °C. For the 42 °C tests, yeast was grown overnight in YMPD medium at 30 °C with shaking at 200 rpm and then diluted to an OD_600_ of 0.1 in fresh YMPD medium and fermented at 42 °C. For the 46 and 50 °C tests, an acclimation period was necessary before the fermentation. For these tests, yeast from a single colony was grown in YMPD medium for 8 h at 42 °C with shaking at 200 rpm before being diluted to an OD_600_ of 0.1 in fresh medium and grown overnight at either 46 or 50 °C. Finally, the cells from the overnight culture were again re-suspended in fresh YMPD medium to an OD_600_ of 0.1 and 0.5 for the fermentations at 46 and 50 °C, respectively, and maintained at the indicated fermentation temperature with shaking at 200 rpm.

*O. polymorpha* was examined at the same temperatures as *K. marxianus* (42, 46, and 50 °C); however, an acclimation period was not necessary for this yeast at any of the temperatures. For these experiments, the yeast was first grown overnight in the YPD medium supplemented with uracil and leucine at 30 °C while shaking at 200 rpm. The next day, cells were diluted to an OD_600_ of 0.1 in fresh YPD with uracil and leucine and fermented at 42, 46, or 50 °C with shaking at 200 rpm.

The third yeast, *S. cerevisiae* strain MAy_6, was examined at a lower temperature range (30, 40, and 42 °C) as this yeast is not thermotolerant. A single colony was first inoculated into SC-Ura-His medium supplemented with 2% glucose and grown overnight at 30 °C with shaking at 200 rpm. The next day, cells were diluted into fresh SC-Ura-His medium to an OD_600_ of 0.1 for tests performed at 30 and 40 °C, and to 0.5 for the 42 °C fermentations.

All fermentations were tested in triplicates and performed in 250-mL Erlenmeyer flasks with an initial culture volume of 50 mL. Flasks were sealed using a plastic cap and by wrapping in parafilm. Samples of 1 mL were obtained at each timepoint and analyzed using HPLC to measure ethanol and glucose concentrations. OD_600_ measurements were obtained using an Eppendorf spectrophotometer (BioSpectrometer basic) for all experiments in this section.

### SSF ethanol production from 0.6% cellulose emulsion and 0.6% MCC solution

The SSF processes were tested for all three yeasts using both a 0.6% cellulose/0.6% hexadecane emulsion as well as a 0.6% MCC solution. Both the emulsion and MCC solution were buffered with 25mM sodium acetate at a pH of 4.8. Cells were initially grown overnight at 30 °C with shaking in their previously described respective media. After this growth, we centrifuged the cultures for 5 min at 234*g* and discarded the supernatant. The cells were then washed with YEP medium (without glucose) and centrifuged again for 5 min at 234*g* to ensure that all residual glucose from the original medium was removed. Next, we inoculated the cells into 25 mL of 16% YEP medium and either emulsion or MCC with 0.6 wt% of cellulose in 125-mL Erlenmeyer flasks. In this step, the *K. marxianus* and *O. polymorpha* (for SSF at 46 °C and 50 °C, respectively) were both inoculated to an OD_600_ of 0.1, whereas the *S. cerevisiae* strain MAy_6 was diluted to OD_600_ values of both 0.1 and 0.5 (for SSF at 30 and 42 °C, respectively). Cellulase enzymes from the Accellerase 1500 cocktail were then added at a concentration of 53 FPU/g cellulose. Before starting the SSF process at the specified temperatures, the flasks were sealed using a plastic cap and parafilm. At each timepoint, 1 mL samples were obtained from each flask and analyzed using HPLC analysis. All fermentations were tested in triplicates.

### Ethanol production from higher cellulose emulsion experiments

Given the overall goal of establishing eSSF as a platform to produce a variety of chemicals from cellulose, we tested *S. cerevisiae*’s SSF ethanol processes at a higher cellulose load to boost titers. We performed these experiments using the MAy_6 *S. cerevisiae* strain in 30 and 42 °C fermentations. Cells were initially grown for 16 h at 30 °C with shaking in 5 mL SC-Ura-His medium supplemented with 2% glucose. After this growth, we centrifuged the cells for 5 min at 234* g*, and discarded the supernatant liquid. The cells were then washed with SC-Ura-His medium (without glucose) and centrifuged again for 5 min at 234* g* to ensure that all residual glucose from the original medium was removed. Cells were then resuspended in 5 mL SC-Ura-His medium without glucose, and inoculated into 12 mL of cellulose medium an OD_600_ of either 0.1 (for 30 °C fermentations) or 0.5 (for 42 °C fermentations). This cellulose medium was composed of 10% (v/v) a solution of 10 × SC-Ura-His with 225 mM sodium acetate buffer (pH = 4.8) and 90% (v/v) of either a 2% cellulose/0.6% hexadecane emulsion or a 2% MCC mixture in deionized water. The Accellerase 1500 cellulase was added to a load of either 26 or 53 FPU/g cellulose, after which the cells were gently mixed in the medium. After the mixture was homogeneous, 1 mL was dispensed into 10 mL screw-top Erlenmeyer flasks (Kemtech America), which were purged with nitrogen for 15 s to remove oxygen from the headspace and finally sealed. The cultures were fermented at either 30 or 42 °C with shaking at 200 rpm, and three flasks were harvested at each timepoint (12, 24, 36, and 48 h). Using the small, sealed flasks prevented oxygen from entering during the fermentation to reduce the consumption of ethanol through respiration. Samples were analyzed for ethanol concentration using HPLC analysis, and all measurements were performed in triplicates.

### Calculating theoretical yields of ethanol in glucose and cellulose

For ethanol fermentations performed using glucose as the feedstock (Fig. 3 and Additional file 1: Figure S2), the theoretical yield of ethanol was calculated using Eq. (1), which assumes that two moles of ethanol can be produced from each mole of glucose. From this value, the percent of theoretical yield can be easily calculated using the measured ethanol concentration:

1 $$\text{Theoretical yield}= \frac{2{{(\text{X}}_{{\text{G}}_{\text{O}}}-{\text{X}}_{\text{G}}) \cdot \text{MW}}_{\text{E}}}{{\text{MW}}_{\text{G}}}$$

where X_GO_ = concentration of glucose at start of fermentation (g/L); X_G_ = concentration of glucose at time of measurement (g/L); MW_E_ = molecular weight of ethanol (g/mol); and MW_G_ = molecular weight of glucose (g/mol).

For the ethanol fermentations performed in cellulose using the mcSSF and eSSF processes (Figs. 4, , Additional file 1: Figure S3), the theoretical yield was evaluated using Eq. (2). The hydrolysis reaction adds one mole of water for each mole of glucose produced, so the equivalent total mass of glucose is 10% greater than the original amount of cellulose, which is accounted for by a factor of 1.1. As in Eq. (1), this formula uses a ratio of two moles of ethanol produced for every mole of glucose consumed:

2 $$\text{Theoretical yield}=\frac{1.1 \cdot \text{ C }\cdot {\text{V}}_{\text{FE}} \cdot 2 \cdot {\text{MW}}_{\text{E}} }{{\text{MW}}_{\text{G}}}$$

where C = cellulose load of the emulsion (g cellulose/mL emulsion), V_FE_ = volume fraction of the total fermentation culture taken up by the emulsion, MW_E_ = molecular weight of ethanol (g/mol), and MW_G_ = molecular weight of glucose (g/mol).

### Quantifying cellulose hydrolysis in SSF ethanol tests

Because yeast consumes the glucose as it is released in the SSF process, the DNS method used to quantify cellulose conversion in the enzymatic hydrolysis tests could not be used to measure conversion in the SSF experiments. We thus estimated the extent of cellulose hydrolysis in the SSF experiments based on the amount of ethanol produced using Eq. (3), which estimates the amount of cellulose that must have been hydrolyzed to achieve the ethanol titers observed. This equation uses a ratio of 1.1 g of glucose per gram of cellulose to account for the mass of the water molecules consumed to form glucose in the hydrolysis reaction, the 1:2 molar ratio of glucose to ethanol, and assumes the same percent of ethanol theoretical yield is achieved in eSSF as in 2% glucose fermentations.

3 $$ \% \text{Conversion}=\frac{\text{Glucose needed to produce observed titers}}{\text{Glucose released if all cellulose is converted}}=\frac{{\text{X}}_{\text{E}} \cdot {\text{MW}}_{\text{G}} }{{2\text{Y }\cdot \text{ V}}_{\text{FE}} \cdot \text{ C }\cdot 1.1}\cdot 100\%$$

where X_E_ = observed concentration of ethanol (mol/mL), MW_G_ = molecular weight of glucose (g/mol), Y = percent theoretical yield when cultured in glucose (calculated from data in Fig. 2), C = cellulose load of the emulsion (g cellulose/mL emulsion), and *V*_FE_ = volume fraction of the total culture taken up by the emulsion (mL emulsion/mL total).

The Y term in this formula, defined as the “percent theoretical yield when cultured in glucose”, is used to account for the fact that some cellulose is inevitably used for cell growth and production of byproducts, rather than for ethanol production. The extent to which the cell converts substrate into biomass and byproducts is assumed to be similar between cellulose and glucose, so this term is calculated from the 2% glucose fermentations at the time of maximum ethanol titer for each yeast and temperature condition tested (using data from Fig. 3 and Additional file 1: Figure S2). These factors as well as the values used to calculate them are summarized for each yeast and temperature (Table 1).

**Table 1:** Maximum ethanol titers and yields from 2% glucose fermentations using different yeasts and conditions

Yeast and temperature condition	Time of maximum ethanol titer	Glucose consumed (g/L)	Ethanol titer (g/L)	% Theoretical yield
*K. marxianus*, 46 °C	12 h	21.05	8.82	82
*O. polymorpha*, 50 °C	48 h	19.01	7.94	82
*S. cerevisiae*, 30 °C	18 h	19.64	8.93	89
*S. cerevisiae*, 42 °C	36 h	14.67	7.29	97

Values listed correspond to the timepoints at which maximum ethanol titers were observed (Fig. 3 and Additional file 1: Figure S2). The percent theoretical yields are used to estimate the extent of cellulose conversion in the eSSF tests (see Eq. (3) in “Methods”)

### Cellulosic isobutanol production using optogenetic SSF process

We performed all SSF isobutanol experiments using the optogenetic *S. cerevisiae* strain YEZ546-2, which is engineered to grow in blue light and produce isobutanol in the dark [50]. A single colony was grown overnight in SC-Ura medium supplemented with 2% glucose at 30 °C with shaking at 200 rpm. This growth took place under blue light, which was supplied by blue light-emitting diode (LED) panels (HQRP New Square 12-inch Grow Light Blue LED 14 W), which had an intensity between 70 and 100 μmol m^−2^ s^−1^ at the distance of the culture, as measured using a Quantum meter with a separate sensor (Model MQ-510 from Apogee Instruments) (with 465 nm max peak spectra). This overnight culture was then diluted into several falcon tubes containing 12 mL of SC-Ura medium at a range of OD_600_ values between 0.2 and 0.8 and regrown for 16 h at 30 °C under blue light. After this second growth, the OD_600_ of the cultures was measured using an Eppendorf spectrophotometer (BioSpectrometer basic), and the tube with an OD_600_ of 9.5 was incubated in the dark for 4 h by being completely covered with aluminum foil. This incubation was performed at 30 °C and with shaking at 200 rpm. After this dark incubation period, the cells were centrifuged for 5 min at 234* g*. The liquid media was discarded, and the cell pellet was resuspended in 12 mL of SC-Ura medium without glucose to wash away any residual glucose from the previous medium. The cells were again centrifuged for 5 min at 234* g*, and the liquid phase was discarded. The cells were then resuspended in a 12 mL mixture of 8.3% SC-Ura (10x), 8.3% sodium acetate buffer (270 mM), and 83.3% of either a 2% cellulose/0.6% hexadecane emulsion or a mixture of 2% MCC in deionized water. Next, 1 mL of the resuspended culture was dispensed into 10 mL screw-top Erlenmeyer flasks (Kemtech America), and different loads of the Accellerase 1500 cellulase cocktail were added to each flask (11, 21, 32, and 53 FPU/g cellulose). The flasks were purged with nitrogen gas for 15 s to remove oxygen in the headspace, sealed, and fermented in the dark at 30 °C for 48 h with shaking at 200 rpm. After fermenting, the cultures were centrifuged, and the supernatants were analyzed for isobutanol concentration using HPLC.

### Measuring analyte concentrations using HPLC

Ethanol, isobutanol, and glucose concentrations for the fermentations were determined by HPLC analysis. Before analyzing the samples, cells and residual cellulose were first separated from the aqueous phase by centrifuging at 234* g* for 10 min at room temperature. The aqueous phase was then transferred into a fresh Eppendorf tube and centrifuged again for 45 min at 13,000* g* at 4 °C. Subsequently, 500 µL of the supernatant was transferred into a glass vial and 5µL injected into an Aminex HPX-87H ion-exchange column (Bio-Rad) to measure ethanol, glucose, and isobutanol concentrations using an Agilent 1260 Infinity instrument (Agilent Technologies). The column was eluted with a mobile phase of 5 mM sulfuric acid at 55 °C and a flow rate of 0.6 mL min^−1^. Ethanol, isobutanol, and glucose measurements were obtained with a refractive index detector (RID) and standard solutions of all three analytes were prepared and analyzed to convert peak areas to concentrations.

## Supplementary Information

**Additional file 1.** Additional figures.

## Data Availability

The datasets generated during the current study are available from the corresponding author on reasonable request.

## Abbreviations

SSF: Simultaneous saccharification and fermentation
eSSF: Emulsified simultaneous saccharification and fermentation
MCC: Microcrystalline cellulose
mcSSF: Microcrystalline cellulose simultaneous saccharification and fermentation
