# Supplementary material for: Cellulosic biofuel production using emulsified simultaneous saccharification and fermentation (eSSF) with conventional and thermotolerant yeasts
Source: Biotechnol Biofuels. 2021 Jul 17;14:157. doi: 10.1186/s13068-021-02008-7 (PMC8285809; doi:10.1186/s13068-021-02008-7)
Supplement: Supplementary file 1 — Additional file 1. Additional figures. [file 13068_2021_2008_MOESM1_ESM.docx]

**
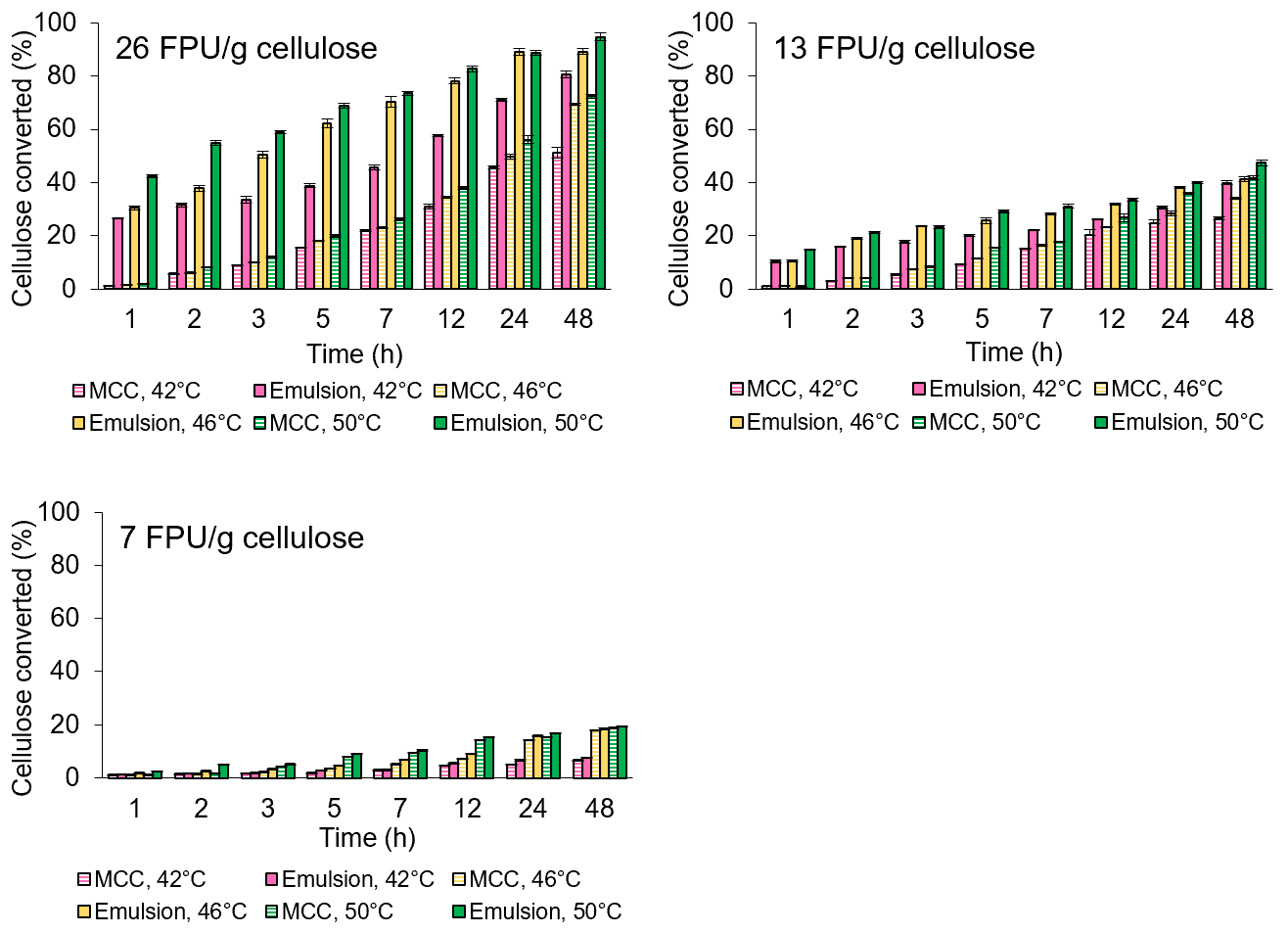
Additional Figures**

**c**

**b**

**a**

**Additional Figure S1.** **Cellulose converted at enzyme loads below 53 FPU/g cellulose.** Cellulose conversion at different temperatures and enzyme loads of **(a)** 26 FPU/g cellulose, **(b)** 13 FPU/g cellulose, and **(c)** 7 FPU/g cellulose using 0.6% cellulose emulsions and microcrystalline cellulose. The emulsion hydrolyzes more effectively than the microcrystalline cellulose, with the possible exception of 7 FPU/g cellulose. Data is shown as the mean values and error bars represent the standard deviation of three replicates.


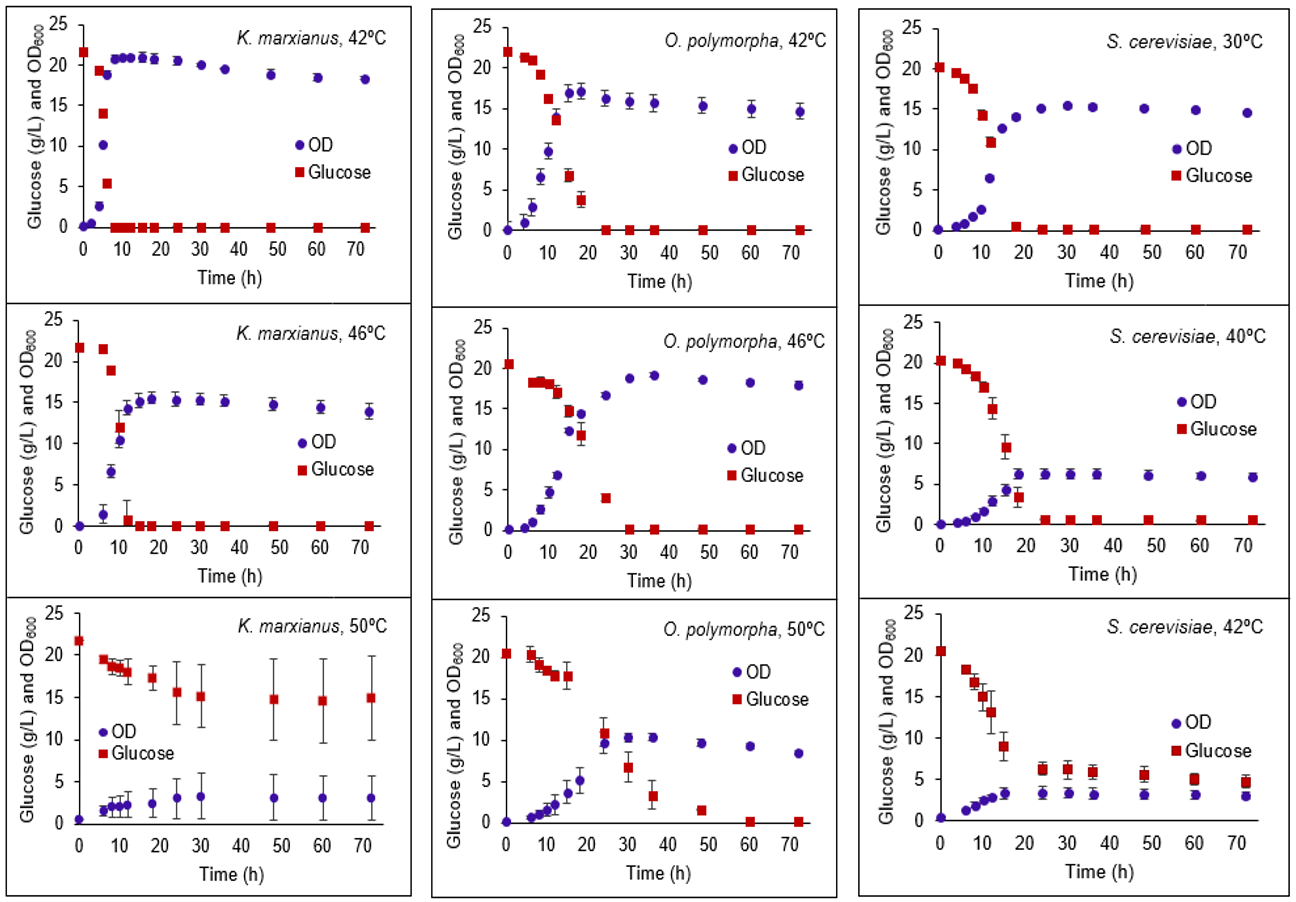


**b**

**c**

**a**

**Additional Figure S2.** **Time course of** **fermentations with *S. cerevisiae* and thermotolerant yeasts.** Cell density (OD_600_) and glucose concentration shown throughout time at different temperatures for **(a)** *K. marxianus*, **(b)** *O. polymorpha*, and **(c)** *S. cerevisiae*. Fermentations initially contained 2% glucose. Data is shown as mean values and error bars represent the standard deviation of three replicates.


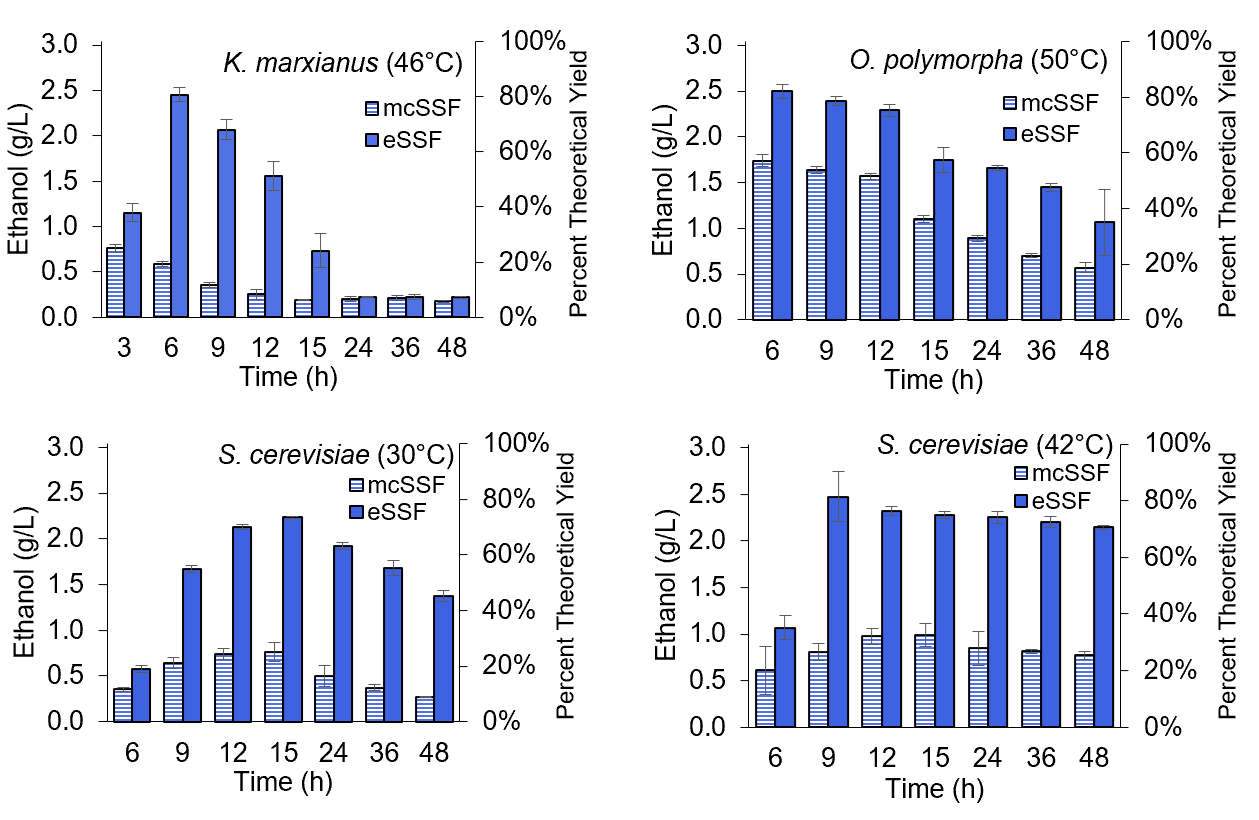
**Additional Figure S3. Time course of ethanol titers obtained from** **mcSSF and eSSF using 0.6% cellulose loads**. The ethanol concentration throughout fermentation is shown for the eSSF and mcSSF processes for **(a)** *K. marxianus* at 42°C, **(b)** *O. polymorpha* at 50°C, and *S. cerevisiae* at **(c)** 30°C and **(d)** 42°C. For all yeasts, the process was performed both with the 0.6% cellulose emulsion (eSSF) as well as a 0.6% MCC mixture (mcSSF). All experiments used an enzyme load of 53 FPU/g cellulose. Data is shown as mean values and error bars represent the standard deviation of three replicates.

**d**

**c**

**b**

**a**


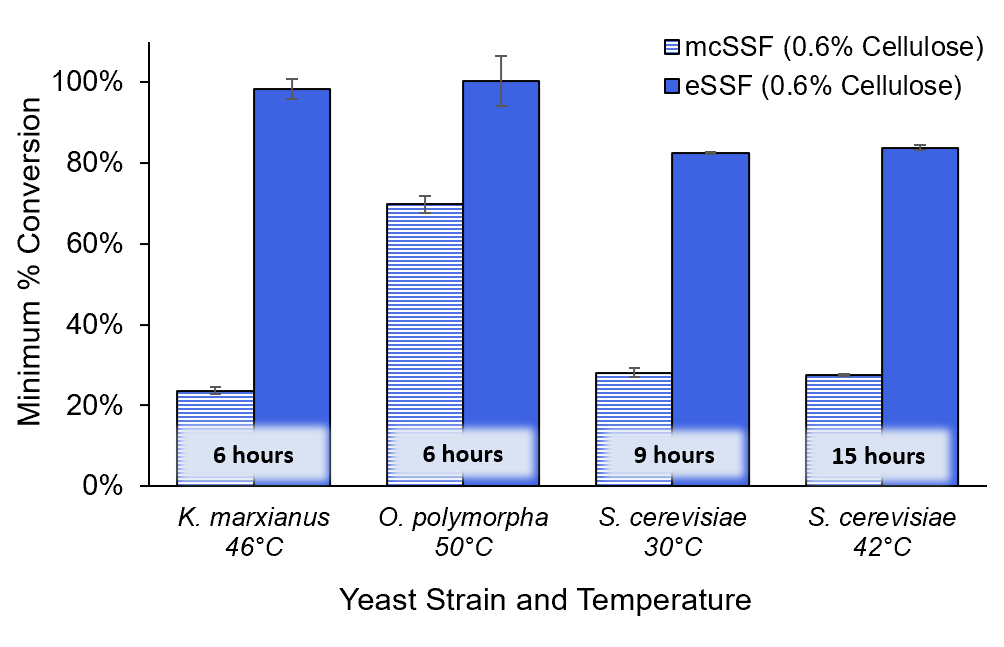
**Additional Figure S4.** **Cellulose percent conversion in mcSSF and eSSF using 0.6% cellulose loads.** The percent conversion of cellulose at the times of peak ethanol titers in the 0.6% cellulose eSSF and mcSSF processes are shown. The percent conversion is estimated based on the amount of cellulose that must have been converted to glucose in order to produce the ethanol titers observed at each condition, assuming the same theoretical yields obtained in glucose fermentations (Table 1 and Equation 1).


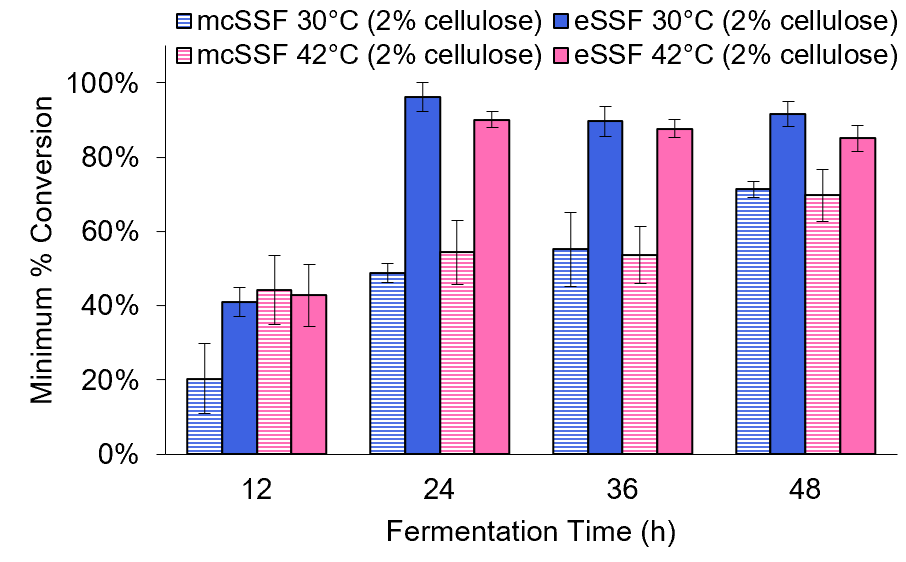
**Additional Figure S5.** **Cellulose percent conversion in mcSSF and eSSF using 2% cellulose loads.** The percent conversion of cellulose at throughout time for 2% cellulose eSSF and mcSSF processes is shown (at an enzyme load of 53 FPU/g cellulose). The conversion is estimated based on the amount of cellulose that must have been converted to glucose in order to produce the ethanol titers observed at each condition, assuming the same theoretical yields obtained in glucose fermentations (Table 1 and Equation 1).
